# Impact of Aldosterone Antagonists on Sudden Cardiac Death Prevention in Heart Failure and Post-Myocardial Infarction Patients: A Systematic Review and Meta-Analysis of Randomized Controlled Trials

**DOI:** 10.1371/journal.pone.0145958

**Published:** 2016-02-18

**Authors:** Hai-Ha Le, Chadia El-Khatib, Margaux Mombled, Frédéric Guitarian, Muaamar Al-Gobari, Mor Fall, Perrine Janiaud, Ivanny Marchant, Michel Cucherat, Théodora Bejan-Angoulvant, François Gueyffier

**Affiliations:** 1 Laboratoire de Biologie et Biométrie Evolutive - Equipe Modélisation des Effets Thérapeutiques, UMR 5558 Université Claude Bernard Lyon1, Lyon, France; 2 Lausanne University Hospital (CHUV), Institute of social & preventive medicine (IUMSP), Lausanne, Switzerland; 3 Escuela de Medicina, Departamento de Pre-clínicas, Universidad de Valparaíso, Valparaíso, Chile; 4 CHRU de Tours, Service de Pharmacologie Clinique, Hôpital Bretonneau, Tours, France; 5 CNRS UMR 7292, Tours, France; 6 Université François-Rabelais, GICC, Tours, France; 7 Service de Pharmacologie Clinique et essais thérapeutiques, Hospices Civils de Lyon, Lyon, France; Hunter College, UNITED STATES

## Abstract

**Background and Objectives:**

Sudden cardiac death (SCD) is a severe burden of modern medicine. Aldosterone antagonist is publicized as effective in reducing mortality in patients with heart failure (HF) or post myocardial infarction (MI). Our study aimed to assess the efficacy of AAs on mortality including SCD, hospitalization admission and several common adverse effects.

**Methods:**

We searched Embase, PubMed, Web of Science, Cochrane library and clinicaltrial.gov for randomized controlled trials (RCTs) assigning AAs in patients with HF or post MI through May 2015. The comparator included standard medication or placebo, or both. Preferred Reporting Items for Systematic Reviews and Meta-Analyses (PRISMA) guidelines were followed. Event rates were compared using a random effects model. Prospective RCTs of AAs with durations of at least 8 weeks were selected if they included at least one of the following outcomes: SCD, all-cause/cardiovascular mortality, all-cause/cardiovascular hospitalization and common side effects (hyperkalemia, renal function degradation and gynecomastia).

**Results:**

Data from 19,333 patients enrolled in 25 trials were included. In patients with HF, this treatment significantly reduced the risk of SCD by 19% (RR 0.81; 95% CI, 0.67–0.98; p = 0.03); all-cause mortality by 19% (RR 0.81; 95% CI, 0.74–0.88, p<0.00001) and cardiovascular death by 21% (RR 0.79; 95% CI, 0.70–0.89, p<0.00001). In patients with post-MI, the matching reduced risks were 20% (RR 0.80; 95% CI, 0.66–0.98; p = 0.03), 15% (RR 0.85; 95% CI, 0.76–0.95, p = 0.003) and 17% (RR 0.83; 95% CI, 0.74–0.94, p = 0.003), respectively. Concerning both subgroups, the relative risks respectively decreased by 19% (RR 0.81; 95% CI, 0.71–0.92; p = 0.002) for SCD, 18% (RR 0.82; 95% CI, 0.77–0.88, p < 0.0001) for all-cause mortality and 20% (RR 0.80; 95% CI, 0.74–0.87, p < 0.0001) for cardiovascular mortality in patients treated with AAs. As well, hospitalizations were significantly reduced, while common adverse effects were significantly increased.

**Conclusion:**

Aldosterone antagonists appear to be effective in reducing SCD and other mortality events, compared with placebo or standard medication in patients with HF and/or after a MI.

## Introduction

Sudden cardiac death (SCD) is defined as unexpected natural death from a cardiac cause within a short time period, generally within one hour from the onset of symptoms, in a person without any prior condition that would appear fatal [1][2]. Patients with previous myocardial infarctions (MI) or cardiac arrest or congestive heart failure (HF) were much more likely to have inducible arrhythmias, considered as a common cause of SCD [3].

The renin-angiotensin aldosterone hormone system’s (RAAS) main function is to maintain the homeostasis of arterial pressure and of extracellular fluids [4]. Dysregulation of this system leads to cardiovascular (CV) disorders including left ventricular remodeling, vasoconstriction/hypertension, and ventricular hypertrophy which may eventually result in SCD [5]. The hormonal cascade is initially induced by a decrease in blood volume which enhances renin secretion into the blood stream, resulting in the production of angiotensin II that is responsible for blood pressure increase via blood vessel constriction and the stimulation of the aldosterone hormone production. Aldosterone in its turn promotes the reabsorption of sodium and water, also leading to an increase in blood pressure [4].

Aldosterone antagonist (AA) inhibits sodium reabsorption and slightly increases water excretion [6]. This group of drugs, including spironolactone, eplerenone, and canrenone among others, is often used in managing chronic and congestive HF [7][8]. Officially, AA treatment is recommended in clinical practice at a low-dose in all patients with a left ventricular ejection fraction (LVEF) < 35% and severe symptomatic HF, i.e. currently New York Heath Association (NYHA) functional class III or IV, in absence of hyperkalemia and significant renal dysfunction, unless contraindicated or not tolerated. It is also recommended in patients suffering acute myocardial infarction (AMI) with LVEF ≤ 40% and developing HF symptoms or having a history of diabetes mellitus, unless contraindicated [9][10].

The benefits of AA in reducing the negative effects of aldosterone hence decreasing death and hospitalization in HF or AMI patients have been demonstrated in four major trials, including RALES (Randomized Aldactone Evaluation Study) [11], EMPHASIS-HF (Eplerenone in Mild Patients Hospitalization and Survival Study in Heart Failure) [12], EPHESUS (Eplerenone Post-AMI Heart Failure Efficacy and Survival Study) [13] and most currently TOPCAT (Treatment of Preserved Cardiac Function Heart Failure with an Aldosterone Antagonist) [14].

Our study aimed to assess the efficacy of AA on SCD, hospitalization admission and several common adverse events in patients with HF or post MI.

## Methods

### Inclusion and exclusion criteria

We included randomized controlled trials (RCTs) comparing spironolactone or eplerenone or canrenoate potassium to placebo or standard treatment. Studies were included if they recruited patients with left ventricular dysfunction HF (NYHA class I to IV) and/or post AMI with Killip scores between I and IV and indicated at least one assessment criteria. Our meta-analysis classified these patients into two corresponding sub-categories: HF and post-MI. The included studies had to report at least one of the following outcomes: SCD, all-cause/CV mortality, all-cause/CV hospitalization and common side effects (hyperkalemia, renal function degradation and gynecomastia).

We excluded studies with a follow-up period < 8 weeks. Trials with inestimable treatment effect (no event in both arms for all criteria) and small sample size (<40 patients/arm) were excluded. The lack of double-blind and/or intention-to-treat analysis of AA efficacy was not an exclusion criterion but was re-examined by sensibility test afterwards.

### Search strategy

The research was conducted systematically from Embase, Medline (Pubmed), Cochrane Library, Web of science and clinicaltrials.gov from 1966 to 31/05/2015 (details of search strategy in S1 App). We searched for studies involving human subjects, clinical trials, RCTs and/or meta-analyses and/or systematic reviews. No language restriction was applied. Preferred Reporting Items for Systematic Reviews and Meta-Analyses (PRISMA) guidelines [15] were followed (S2 App).

Study screening and analyzing through titles and abstracts was performed independently by several investigators in different periods (HHL, MM, CK, TA, FG), according to the pre-specified selection criteria. Data were extracted independently and compared afterwards. The latest screening and data extraction (through May 2015) were conducted independently by two investigators (HHL & MM) with kappa statistics (S3 App). Cochrane bias criteria [16] were used to evaluate the overall quality of the articles. An included trial was considered as of high quality if all its risks of bias were low. Disagreements were discussed and decisions were made through consensus. A third party (FG) was involved when necessary. The following information was extracted from the studies: the first author or study name, year of publication, baseline patient characteristics, intervention and related outcomes. Besides database searching, reference lists of all included studies, meta-analyses and reviews were manually searched for further potential trials and/or information validation.

### Outcomes assessment

The primary endpoints were SCD, total mortality and CV mortality at the end of the follow-up duration. Secondary outcomes were hospitalization (from all causes and CV causes) and adverse reaction events (hyperkalemia, renal function degradation and gynecomastia) by AAs.

### Statistical analysis

Kappa statistic was calculated for agreement ratio between two latest reviewers (HHL & MM) (S3 App). We extracted aggregate data, number of events and number of patients in each subgroup from included studies, using fixed-effect and random-effect models to pool the data. Results were reported as relative risk (RR) at 95% confidence intervals (CI) using the Mantel and Haenszel method for the fixed-effect model [17] or the DerSimonian and Laird method for the random-effect model [18]. When similar outcomes were obtained by both methods, we only reported the random-effect results to cover possible heterogeneity as several pharmacologic drugs and different patients were included.

Heterogeneity across studies was estimated using I^2^ test [18]. I^2^ values of 25%, 50%, and 75% correspond to low, moderate, and high levels of heterogeneity [19]. Meta-analysis results were considered only if the I^2^ value was below 75%. Potential existence of publication bias was assessed in both subgroups at each criterion of outcome by funnel plots and verified by the Egger tests [20] using odds ratio (OR) since firm guidance for RR is not yet available [21]. Sensitivity analysis was carried out for each outcome measure to evaluate the contribution of each study to the pooled estimate by excluding important trials/ lack of blinding trials/ lack of intention-to-treat analysis trials at one time and recalculating the combined RR for the remaining studies. Statistical testing was two-tailed, with statistical significance declared at 5%. All analyses were performed using RevMan (version 5.3) and R (version 3.2.2) softwares.

## Results

### Search results

**Fig 1:**
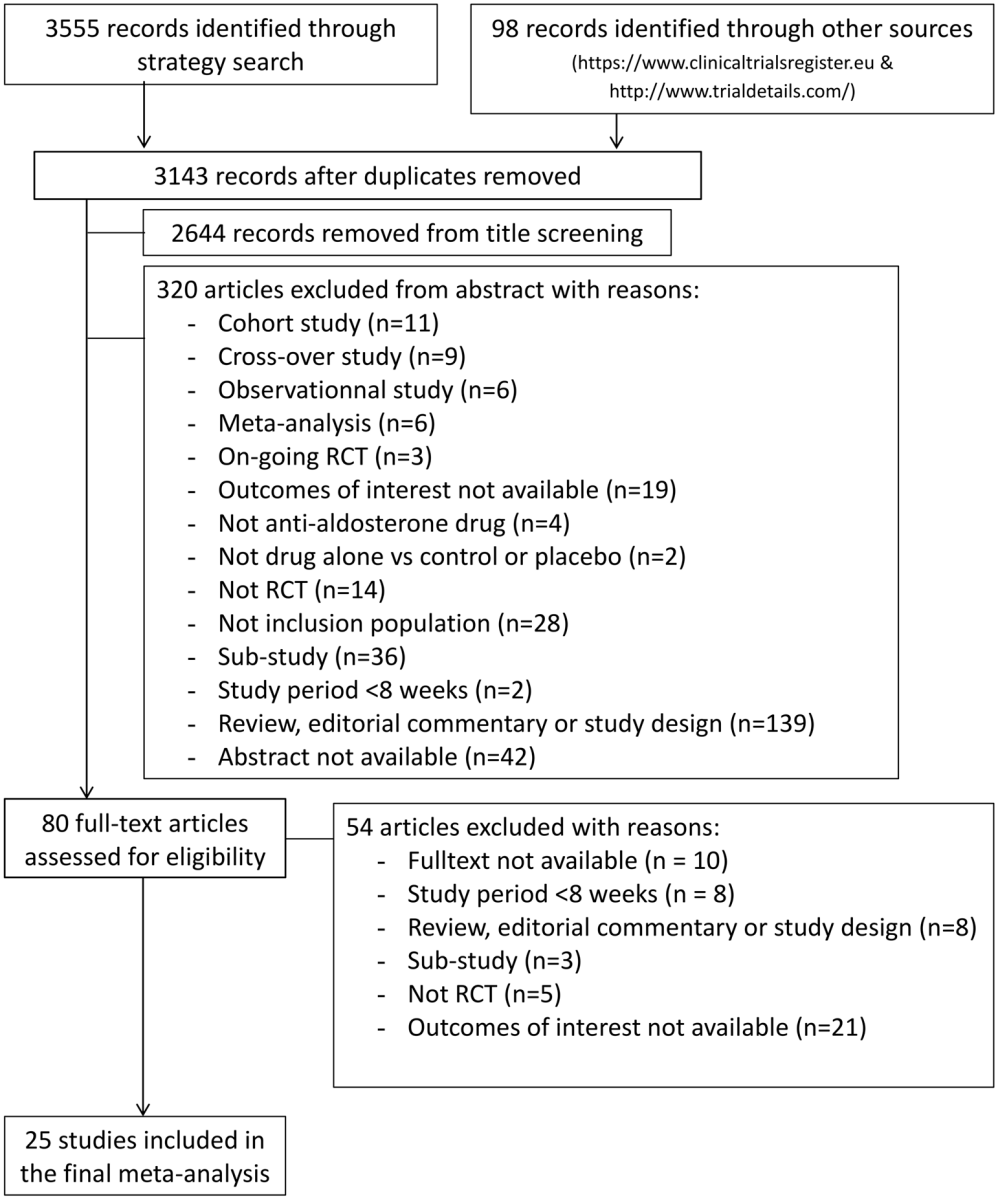
Study flowchart for the selection process of the final included trials.

Our search through Embase, Medline (Pubmed), Cochrane Library, Web of science, clinicaltrials.gov and other sources (www.clinicaltrialsregister.eu & www.trialdetails.com) returned a total of 3653 studies. After elimination of duplicates, 3143 studies were retained for evaluation. Through screening of titles and abstracts, 2644 and 320 irrelevant studies were respectively excluded, respectively. Following full manuscript review of the remaining 80 studies, 54 additional ones were excluded: full-text not available (n = 10) (correspondences to authors were made but we have not received positive responses), study period <8 weeks (n = 8), review, editorial commentary or study design (n = 8), sub-study (n = 3), not RCT (n = 5), and outcomes of interest not available (n = 21). Finally, 25 studies satisfying all selection criteria were included in this meta-analysis (Fig 1). The kappa statistic indicated a subtidal agreement good at 0.75 (IC 95% CI, 0.49–1.02; p = 0.0005) (S3 App).

The quality of evidence of included studies was relatively high: 100% of low risk for selection, attrition and reporting biases, 70% of low risk for performance bias and >85% of low risk for detection bias (S1 Fig).

### Study characteristics

In total, 25 RCTs [11],[13–14],[22–32],[33–43] were selected in this meta-analysis, which enrolled a total of 19333 patients (9750 for AA arm and 9583 for control/placebo arm). The mean follow-up duration was 12.42 months (1.04 year). All trials were placebo controlled except three trials [22][23][24] which applied routine treatment. Nine trials [25][26][27][28][13][29][30][31][24] assessed the effect of AAs in post-AMI patients with left ventricular dysfunction; while the other trials recruited HF patients. Duration of follow-up varied from 3 to 44 months. Spironolactone was the most commonly used AAs (15 studies), followed by eplerenone (7 studies) and canrenone (3 studies) (Table 1). The risk of bias of included trials was presented in S1 Table and S1 Fig.

**Table 1:** Main characteristics of included studies.

*Studies*, *(abbreviation name)*, *year of publication*	*Patients; duration (follow-up); countries*	*Comparison*	*Study design*, *intention to treat analysis (ITTA)*	*Number of randomized patients (excluded during follow-up)*	*Mean age (SD)*	*Male sex (%)*	*Ischemic etiology (%)*	*Ejection fraction (%)*
*Boccanelli et al*. *2009 (AREA-in-HF)* [35]	HF; 12 months; Italy	Canrenone 25 mg (titrated to 50 mg/day) vs. Placebo	DB; without ITTA	231(43)/ 236(42)	62.3(9.5)/ 62.7(9.5)	82/85	51.1/ 52.1	39.9(8.6)/ 39.7(8.6)
*Chan et al*. *2007* [56]	HF; 12 months; China	Spironolactone 25 mg/day + candesartan vs. Placebo + candesartan	DB; with ITTA	23(0)/25(0)	61.4(12.3)/ 65(0.6)	87/80	47.8/ 64.0	26(2)/28(2)
*Cicoira et al*. *2002* [22]	HF; 12 months; Italy	Spironolactone 25 mg (titrated to 50 mg/day) vs. Routine treatment	Open label, without ITTA	54(7)/52(6)	62.5(7.9)/ 61.7(9.8)	85/88	65/63	33(7)/34(7)
*Deswal et al*. *2011(RAAM-PEF)* [36]	HF; 6 months; USA	Eplerenone 25 mg (titrated to 50 mg/day) vs. Placebo	DB; without ITTA	23(2)*/23(0)*	72.2(9.8)*/ 68.7(9.1)*	95*/91*	NR/NR	62.1(5)*/ 62.5(7.5)*
*Di Pasquale et al*. *2005* [25]	MI; 6 months; Italy	Canrenoate IV 1 mg/h then 25 mg PO/day + Captopril vs. Placebo + Captopril	DB; without ITTA	341(33)/ 346(30)	62.6(6)/ 62.8(5)	71/71	100/100	NR/NR
*Edelmann et al*. *2013 (Aldo-DHF)* [32]	HFPEF; 12 months; Germany &Austria	Spironolactone 25 mg/day vs. Placebo	DB; with ITTA	213(0)/209(0)	67(8)/67(8)	48/47	NR/NR	67(8)/68(7)
*Gao et al*. *2007* [57]	HF; 6 months; China	Spironolactone 20 mg/day vs. Placebo	DB, with ITTA	58(0)/58(0)	55(13)/54(12)	64/66	50/52	42(11)/43(10)
*Kayrak et al*. *2010* [26]	AMI; 6 months; Turkey	Spironolactone 25 mg/day vs. Routine treatment	Open label, without ITTA	71(16)/71(16)	55.3(10)*/ 57.2(11)*	18*/26*	100/100	50.5(8.3)*/ 49.5(8)*
*Mak et al*. *2009* [23]	DHF; 12 months; Ireland	Eplerenone 25 mg (titrated to 50 mg/day) vs. Routine treatment	Open label, without ITTA	24(1)/20(3)	80(7.7)/ 79(7.9)	38/55	NR/NR	63(9.0)/64(9.6)
*Modena et al*. *2001* [27]	MI; 12 months; Italy	Potassium canrenoate 50 mg/day vs. Placebo	NR, with ITTA	24(0)/22(0)	59(10)/62(13)	71/77	100/100	47(6)/46(5)
*Montalescot et al*. *2014 (REMINDER)*[28]	MI; 10.5 months; International (11 countries)	Eplerenone 25 mg (titrated to 50 mg/day) vs. Placebo	DB; with ITTA	506(82)/506(79)	58.5(10.8)/ 57.8(11.0)	83/80	100/100	NR/NR
*Pitt et al*. *2014 (TOPCAT)* [14]	HF; 3.3 years; International (6 countries)	Spironolactone (15 to 45 mg/day) vs. Placebo	DB; with ITTA	1722(0)/ 1723(0)	68.7(median) range 61.0–76.4/ 68.7(median) range 60.7–75.5	NR/NR	NR/NR	56(median) range 51–61/ 56(median) range 51–62
*Pitt et al*. *2003 (EPHESUS)* [13]	LVD after MI; 16 months (range 0–33); International (37 countries)	Eplerenone 25 mg (titrated to 50 mg/day) vs. Placebo	DB; with ITTA	3319(0)/ 3313(0)	64(11)/64(12)	72/70	100/100	33(6)/33(6)
*Pitt et al*. *1999 (RALES)* [11]	HF; 24 months; International (15 countries)	Spironolactone 25 mg (titrated to 50 mg/day) vs. Placebo	DB; with ITTA	822(0)/841(0)	65(12)/65(12)	73/73	55/54	25.6(6.7)/ 25.2(6.8)
*Taheri et al*. *2012* [37]	CHF; 6 months; Iran	Spironolactone 25 mg/day vs. Placebo	DB; without ITTA	9(2)/9(3)	50.7(17.4)/ 57.2(13.1)	55/55	NR or 0/ NR or 0	26.6(8.3)/ 31.1(10.5)
*Taheri et al*. *2009* [38]	HF; 6 months; Iran	Spironolactone 25 mg/day vs. Placebo	DB; without ITTA	8(3)/8(2)	59.5(6.5)/ 56.8(9.3)	63/75	NR or 0/ NR or 0	31.3(8.7)/ 33.8(9.2)
*The RALES Investigators* [58]	HF; 3 months; International	Spironolactone 12.5, 25, 50, 75 mg/day (4 groups) vs. Placebo	DB; with ITTA	174(0)/40(0)	63/61(12)	79/83	NR/NR	NR/NR
*Udelson et al*. *2010* [39]	HF; 9 months; USA (multicenter)	Eplerenone 25 mg (titrated to 50 mg/day) vs. Placebo	DB; without ITTA	117(13)/109(20)	63.3(12.2)/ 62.0(12.9)	84/84	60/61	26.2(0.6)/ 27.0(0.6)
*Uzunhasan et al*. *2009* [29]	AMI; 6 months; Turkey	Spironolactone 50 mg/day vs. Placebo	DB; with ITTA	41(0)/41(0)	52(10)/52(10)	79/71	NR/NR	47/44
*Vatankulu et al*. *2013* [30]	AMI; 6 months; Turkey	Spironolactone 12.5 & 25 mg/day (2 groups) vs. Routine treatment	Open label; with ITTA	104(0)/56(0)	56/57(11)	84/80	100/100	NR/NR
*Vizzardi et al*. *2013* [34]	CHF; 44 ± 16 months; Italy	Spironolactone 25 mg (titrated to 100 mg/day) vs. Placebo	SB; without ITTA	65(5)/65(1)	61(14.7)/ 65(17.4)	NR/NR	NR/NR	34.5(6.8)/ 37.7(11)
*Vizzardi et al*. *2010* [59]	HF; 6 months; Italy	Spironolactone 25 mg (titrated to 100 mg/day) vs. Placebo	SB; with ITTA	79(0)/79(0)	61(13)/58(13)	84/82	NR/NR	35.2(0.7)/ 35.4(1.0)
*Weir et al*. *2009* [31]	MI; 5.5 months; UK	Eplerenone 25 mg (titrated to 50 mg/day) vs. Placebo	DB; without ITTA	50(4)/50(3)	61.0 12.0)*/ 56.8(12.0)*	74*/80*	100/100	35.2(3.9)*/ 32.3(4.8)*
*Wu et al*. *2013* [24]	AMI; 12 months; China	Spironolactone 20 mg/day vs. Routine treatment	Open label; without ITTA	308(46)/308(42)	59.8(11.7)*/ 59.9(10.3)*	74*/72*	100/100	NR/NR
*Zannad et al*. *2011 (EMPHASIS-HF)* [33]	HF; 21 months; International	Eplerenone 25 mg (titrated to 50 mg/day) vs. Placebo	DB; with ITTA	1364(0)/ 1373(0)	68.7(7.7)/ 68.6(7.6)	77/78	70/68	26.2(4.6)/ 26.1(4.7)

The results are shown according to the mean (SD), except for additional explanation in exceptional cases. BD: double blind; ITTA: intention to treat analysis; HF: Heart failure; DHF: Diastolic heart failure; CHF: congestive heart failure; HFPRE: Heart failure with preserved ejection fraction; MI: Myocardial infarction; LVD: Left Ventricular Dysfunction; IV: Intra-venous; DB: Double blind; SB: Single blind; NR: not reported; AREA-in-HF: Aldosterone Receptor Antagonists improve outcome in severe Heart Failure; RAAM-PEF: Randomized Aldosterone Antagonism in Heart Failure with Preserved Ejection Fraction; Aldo-DHF: Aldosterone Receptor Blockade in Diastolic Heart Failure; TOPCAT: Treatment of Preserved Cardiac Function Heart Failure With an Aldosterone Antagonist; EPHESUS: Eplerenone Post-Acute Myocardial Infarction Heart Failure Efficacy and Survival Study; RALES: Randomized Aldactone Evaluation Study; EMPHASIS-HF: Eplerenone in Mild Patients Hospitalization and Survival Study in Heart Failure.

^(^*^)^ For only the patients included in final analyses.

### Baseline patient characteristics

Most trials included elderly people with mean age ranged from 50–80 years (Table 1). Most of studies consisted dominantly male participants, except two trials [26][23] where more women were recruited and the trial of Edelmann *et al*. [32] which had a relatively equal sex ratio. All trials were restricted to patients without renal dysfunction (kalemia <5.5 mmol/l and creatinine < 2.5 mg/dL) (Table 2).

**Table 2:** Main criteria for patients’ eligibility in the included studies.

*Studies*	*NYHA class*	*Killip class*	*Creatinine (mg/dL or other units)*	*Serum potassium (mmol/L)*	*Ejection fraction (%)*
*Boccanelli et al*. *2009 (AREA-in-HF)* [35]	II	NR	≤2.5	≤5.0	≤45
*Chan et al*. *2007* [56]	II to III	NR	≤200 μmol/l	≤5.0	<40
*Cicoira et al*. *2002* [22]	NR	NR	≤150 μmol/l	≤5.0	≤45
*Deswal et al*. *2011(RAAM-PEF)* [36]	II to III	NR	≤2.5	≤5.0	≥50
*Di Pasquale et al*. *2005* [25]	NR	I to II	<2.0	<5.0	NR
*Edelmann et al*. *2013* [32]	II to III	NR	NR	<5.1	≥50
*Gao et al*. *2007* [57]	II to IV	NR	<2.5	<5.5	<45
*Kayrak et al*. *2010* [26]	NR	I to II	≤2.0	≤5.0	≥40
*Mak et al*. *2009* [23]	IV	NR	≤200 μmol/l	NR	≥45
*Modena et al*. *2001* [27]	NR	I to III	≤2.5	NR	NR
*Montalescot et al*. *2014 (REMINDER)* [28]	NR	NR	≤2.5	NR	≤40
*Pitt et al*. *2014 (TOPCAT)* [14]	I to IV	NR	<2.5	≤5.0	≥45
*Pitt et al*. *2003 (EPHESUS)* [13]	I to IV	NR	≤2.5	≤5.0	≤40
*Pitt et al*. *1999(RALES)* [11]	III to IV	NR	≤2.5	≤5.0	≤35
*Taheri et al*. *2012* [37]	III to IV	NR	NR	<5.5	≤45
*Taheri et al*. *2009* [38]	III to IV	NR	NR	≤5.5	≤45
*The RALES Investigators* [58]	III to IV	NR	≤2.0	<5.5	≤35
*Udelson et al*. *2010* [39]	II to III	NR	NR	≤5.5	≤35
*Uzunhasan 2009* [29]	NR	I to II	<2.5	≤5.0	NR
*Vatankulu et al*. *2013* [30]	NR	I to II	≤2.0	<5.5	≥40
*Vizzardi et al*. *2013* [34]	I to II	NR	NR	≤5.0	<40
*Vizzardi et al*. *2010* [59]	I to II	NR	≤2.5	≤5.0	≤40
*Weir et al*. *2009* [31]	NR	I to II	≤2.5	≤5.0	<40
*Wu et al*. *2013* [24]	NR	I to III	≤2.5	≤5.0	NR
*Zannad et al*. *2011 (EMPHASIS-HF)* [33]	II	NR	NR	≤5.0	≤35

NYHA: New York Heath Association; ND: Not Defined; NR: Not Reported; 221 μmol/l ~ 2.5 mg/dL.

### Primary outcomes

#### Sudden cardiac death

In the 25 included articles, six accounting for 8301 subjects (4132 used AAs and 4169 received placebo/control) reported SCD events in patients with HF. In the follow-up duration, the SCD rate in HF patients was 4.89% (n = 202/4132) in those treated with AAs, compared with 6.09% (n = 254/4169) in those treated with placebo/control. In post-MI patients, SCD was reported only in the EPHESUS trial [13] at the rates of 4.88% (n = 162/3319) and of 6.07% (n = 201/3313) in groups receiving AAs and placebo, respectively.

**Fig 2:**
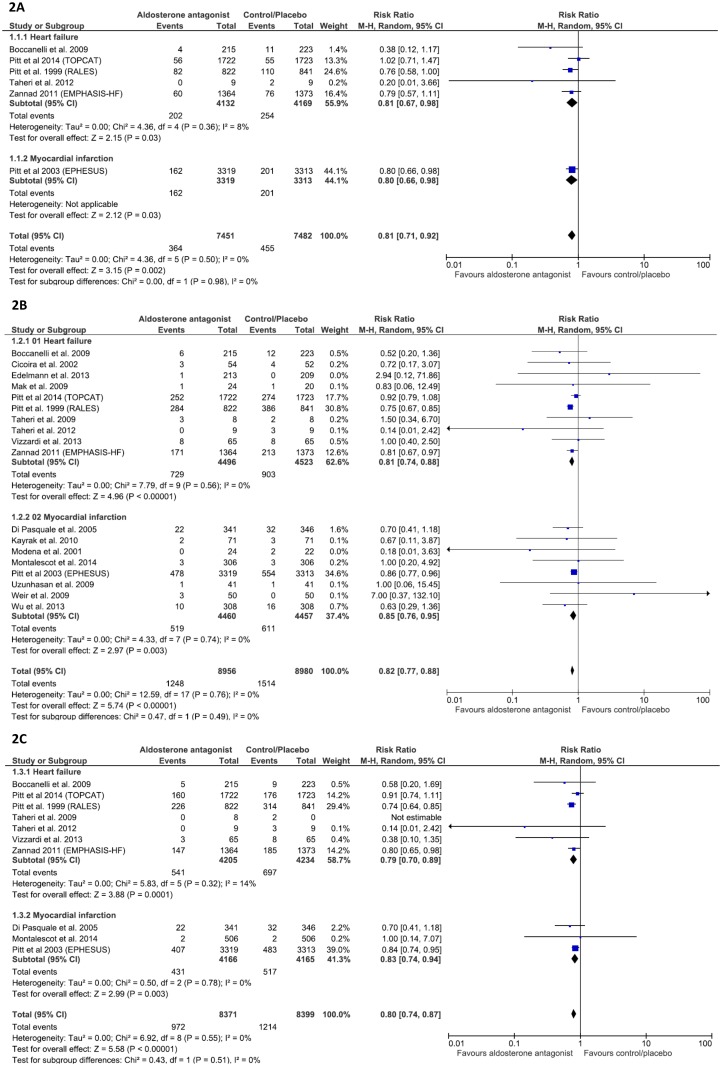
Efficacy of aldosterone antagonist compared with control for the prevention of (A) Sudden death, (B) All-cause mortality, and (C) Cardiovascular death in patients with heart failure or myocardial infarction.

There was a significant reduction of SCD rate with AAs in patients with HF (19% SCD reduction; RR 0.81; 95% CI, 0.67–0.98; p = 0.03) or with post-MI left ventricular dysfunction (20% SCD reduction; RR 0.80; 95% CI, 0.66–0.98; p = 0.03). In total, the SCD rate was 4.88% (n = 364/7451) in those treated with AAs compared with 6.08% (n = 455/7482) in those treated with placebo/control (19% SCD reduction; RR 0.81, 95% CI, 0.71–0.92; p = 0.002) without any evidence of statistical heterogeneity (I^2^ = 0%) (Fig 2A).

#### All-cause mortality

All-cause mortality rate in patients with HF were 16.21% (n = 729/4496) in those treated with AAs and 19.96% (n = 903/4523) in those assigned to placebo/control (RR 0.81, 95% CI, 0.74–0.88, p<0.00001) through the follow-up duration. The corresponding numbers in the sub-group of MI were 11.64% (n = 519/4460) and 13.71% (n = 611/4457), respectively, with 15% reduction (RR 0.85; 95% CI, 0.76–0.95, p = 0.003). Altogether, there were 1248/8956 (13.93%) and 1514/8980 (16.86%) deaths from all causes, respectively, observed in treatment and placebo arms with a general reduction rate of 18% (RR 0.82; 95% CI, 0.77–0.88, p<0.00001). Heterogeneity was not found in each sub-group (consisting 10 and 8 trials, respectively) and in the whole population (all I^2^ = 0%) (Fig 2B).

#### Cardiovascular mortality

In the follow-up duration, CV mortality rate was 17.03% (n = 541/4205) in those treated with AAs and 22.54% (n = 697/4234) in those received placebo in the HF subgroup, resulting in a reduction rate of 21% (RR 0.79; 95% CI, 0.70–0.89, p<0.00001). In the MI subgroup, the efficacy of AAs was demonstrated by a reduction of 17% (RR 0.83; 95% CI, 0.74–0.94, p = 0.003) of CV mortality in treated patients compared with those receiving placebo (431/4166 vs 517/4165 deaths, respectively). AAs contributed a general reduction of 20% for the two categories of patients (RR 0.80; 95% CI, 0.74–0.87, p<0.00001) (Fig 2C).

Generally, there were likely no heterogeneity found in SCD, all-cause mortality and CV mortality (all I^2^ = 0%), regarding both categories of patients.

### Secondary outcomes

#### All-cause hospitalization

**Fig 3:**
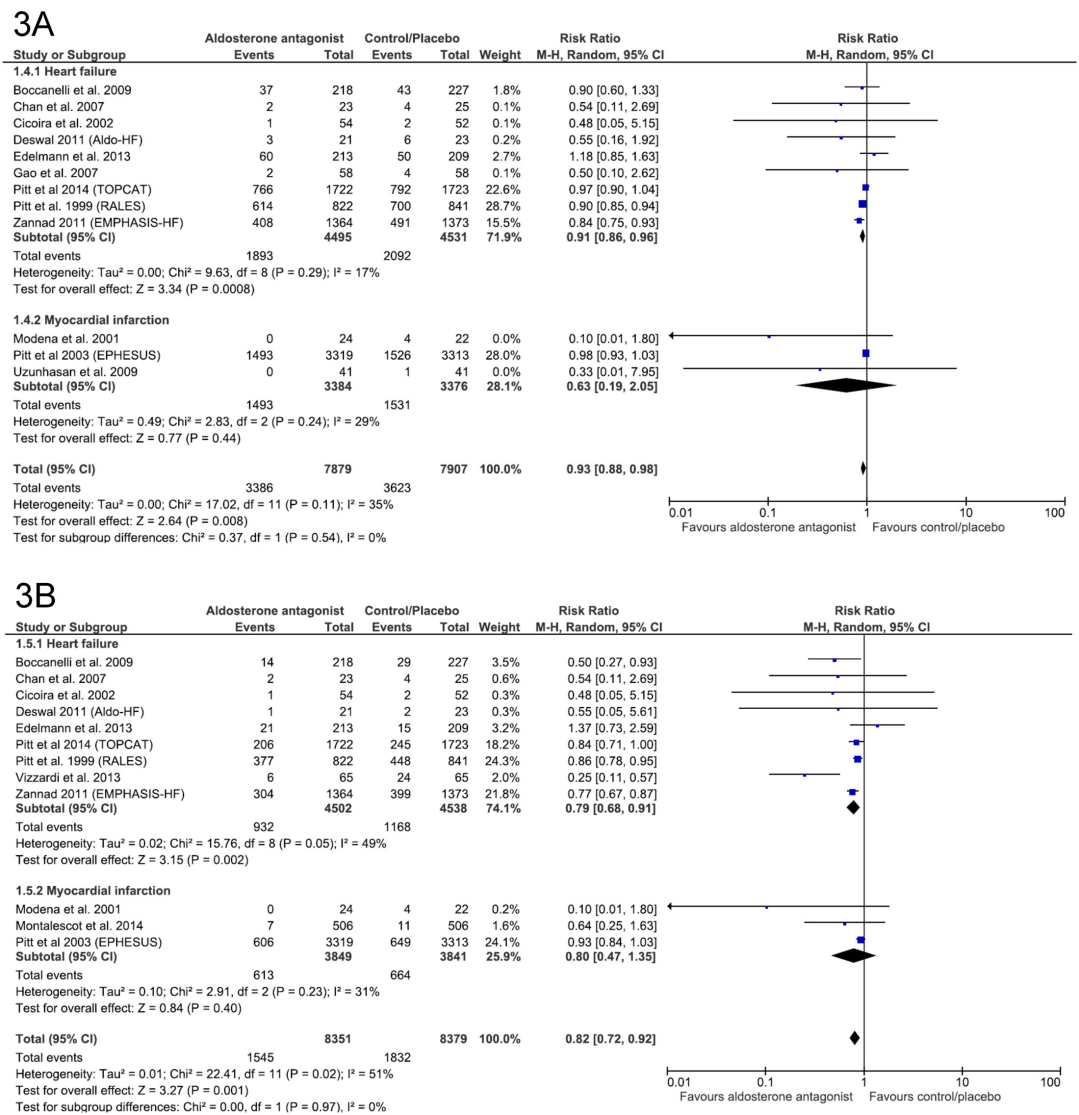
Efficacy of aldosterone antagonist compared to control for the prevention of (A) All-cause hospitalization and (B) Cardiovascular hospitalization in patients with heart failure or myocardial infarction.

Relative risk reductions in all-cause hospitalization rate by AAs compared with placebo/control were 9% in HF patients (RR 0.91; 95% CI, 0.86–0.96; p = 0.0008) and 37% in post-MI patients (RR 0.63; 95% CI, 0.19–2.05; p = 0.44). In overall analysis, the results showed a significant decrease of 7% of all–cause hospitalization in patients receiving AAs compared with those taking placebo/control (RR 0.93; 95% CI, 0.88–0.98; p = 0.008) (Fig 3A). However, heterogeneity was likely considerable (I^2^ = 17%, 29% and 35% respectively).

#### Cardiovascular hospitalization

In patients with HF, a significant relative risk reduction of 21% for CV hospitalization was observed in those assigned to AAs, compared with placebo/control (RR 0.79; 95% CI, 0.68–0.91; p = 0.002). In patients with MI, the corresponding value was 20% but not significant (RR 0.80; 95% CI, 0.47–1.35; p = 0.44). An analysis for both subgroups showed a relative risk reduction of 18% (RR 0.82, 95% CI, 0.72–0.92; p = 0.001) (Fig 3B). However, heterogeneity detected was moderate (I^2^ = 49%, 31% and 51% respectively).

### Adverse reactions

**Fig 4:**
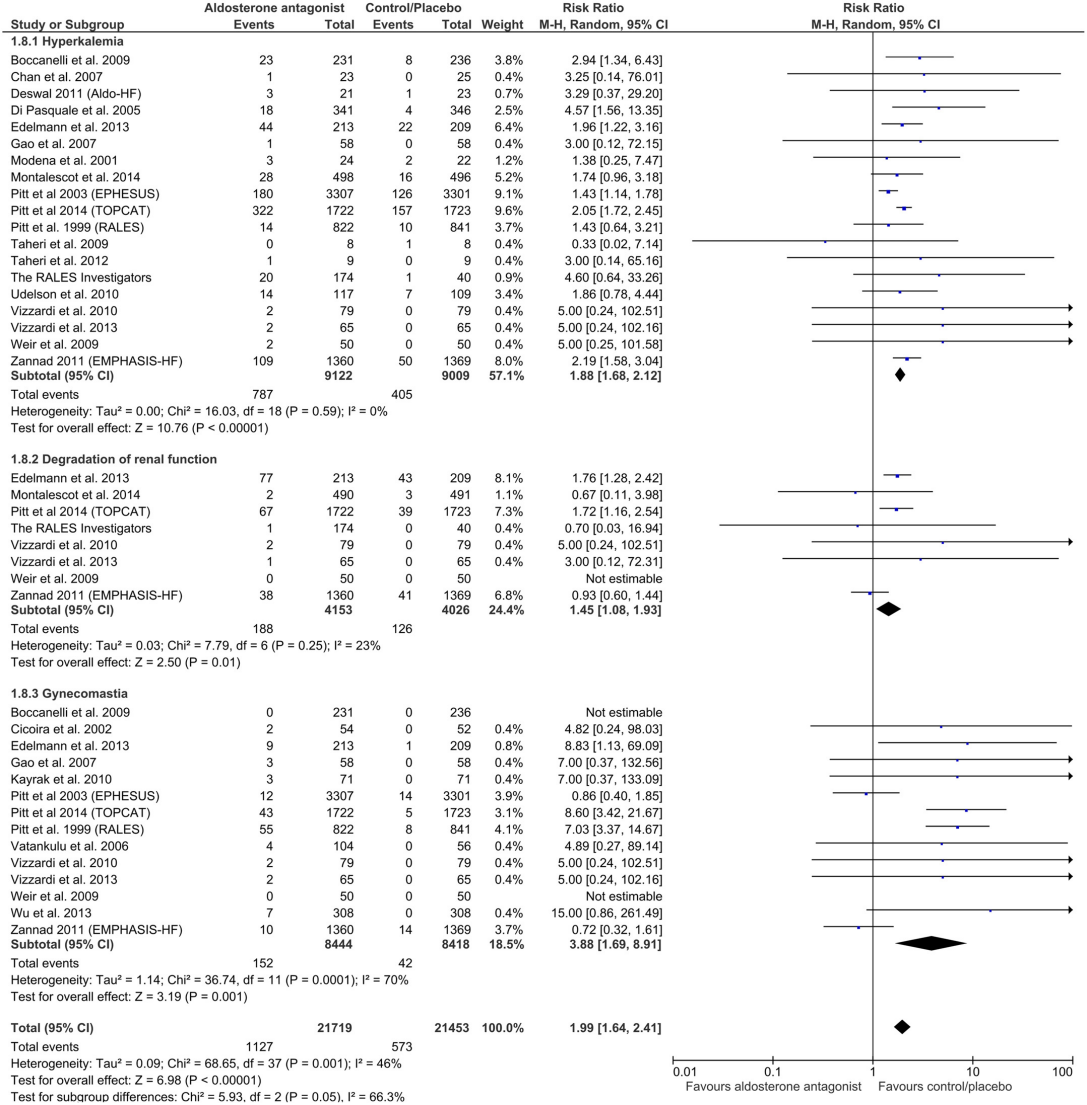
Incidences of adverse effects (hyperkalemia, degradation of renal function and gynecomastia) under aldosterone antagonist treatment, compared with control/placebo group, in patients with heart failure or myocardial infarction.

Hyperkalemia, worsening renal function and gynecomastia were the main observed side effects of AAs in the 25 included studies, as compared to placebo or control. In general, the incidence of all considered adverse events significantly doubled in patients treated with AAs, compared to those receiving placebo or reference therapy. Corresponding RRs were 1.88 (Cl 95%, 1.68–2.12, p<0.00001) for hyperkalemia; 1.45 (CI 95%,1.08–1.93, p = 0.01) for degradation of renal function; 3.88 (CI 95%, 1.69–8.91, p = 0.001) for gynecomastia and 1.99 (95% CI, 1.64–2.41; p<0.00001) for all considered side-effects in general, with remarkably various heterogeneities found among the subgroups (0%, 23%, 70% and 46% respectively) (Fig 4). Exceptions appeared for the two big RALES and EMPHASIS-HF trials [11][33], where interestingly enough, patients in the placebo groups had slightly higher rate of gynecomastia (RALES and EMPHASIS-HF) and of renal function degradation (EMPHASIS-HF).

### Publication bias

**Fig 5:**
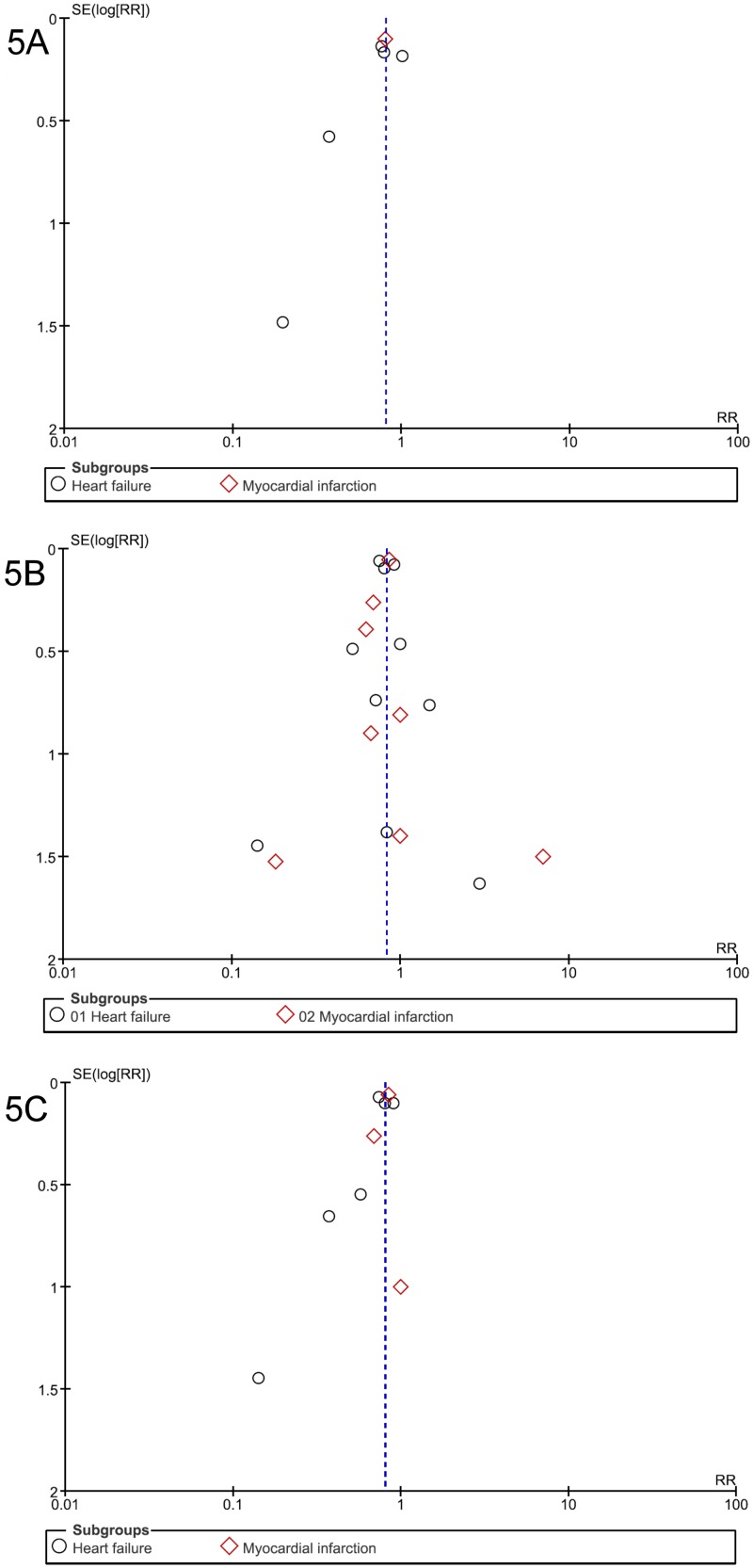
Funnel plot of standard error (log odds ratio) by odds ratio to evaluate publication bias for effect of aldosterone antagonist treatment in preventing (A) Sudden death, (B) All-cause mortality, and (C) Cardiovascular mortality in patients with heart failure or myocardial infarction.

**Fig 6:**
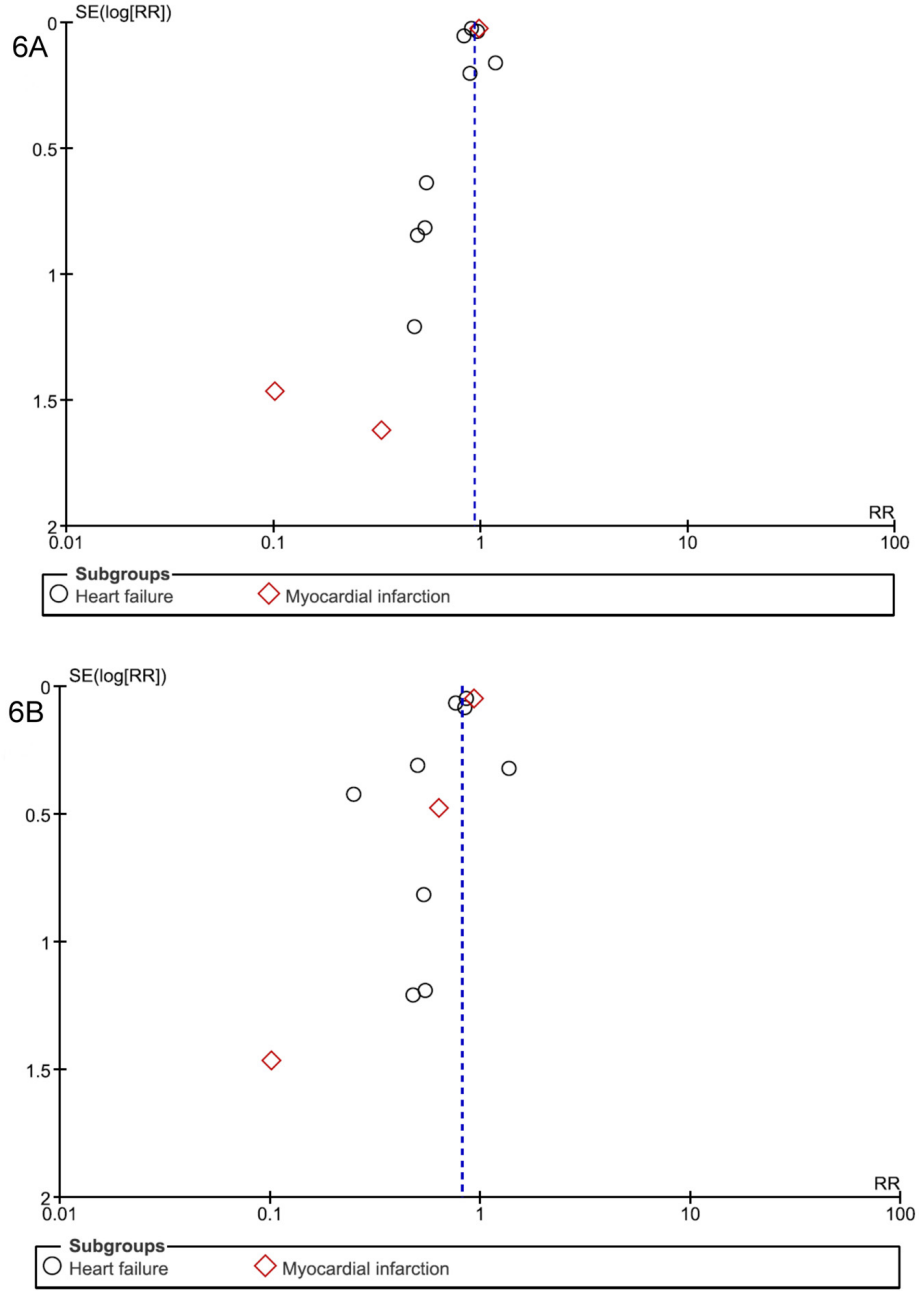
Funnel plot of standard error (log odds ratio) by odds ratio to evaluate publication bias for effect of aldosterone antagonist treatment in preventing (A) All-cause hospitalization and (B) Cardiovascular hospitalization in patients with heart failure or myocardial infarction.

**Fig 7:**
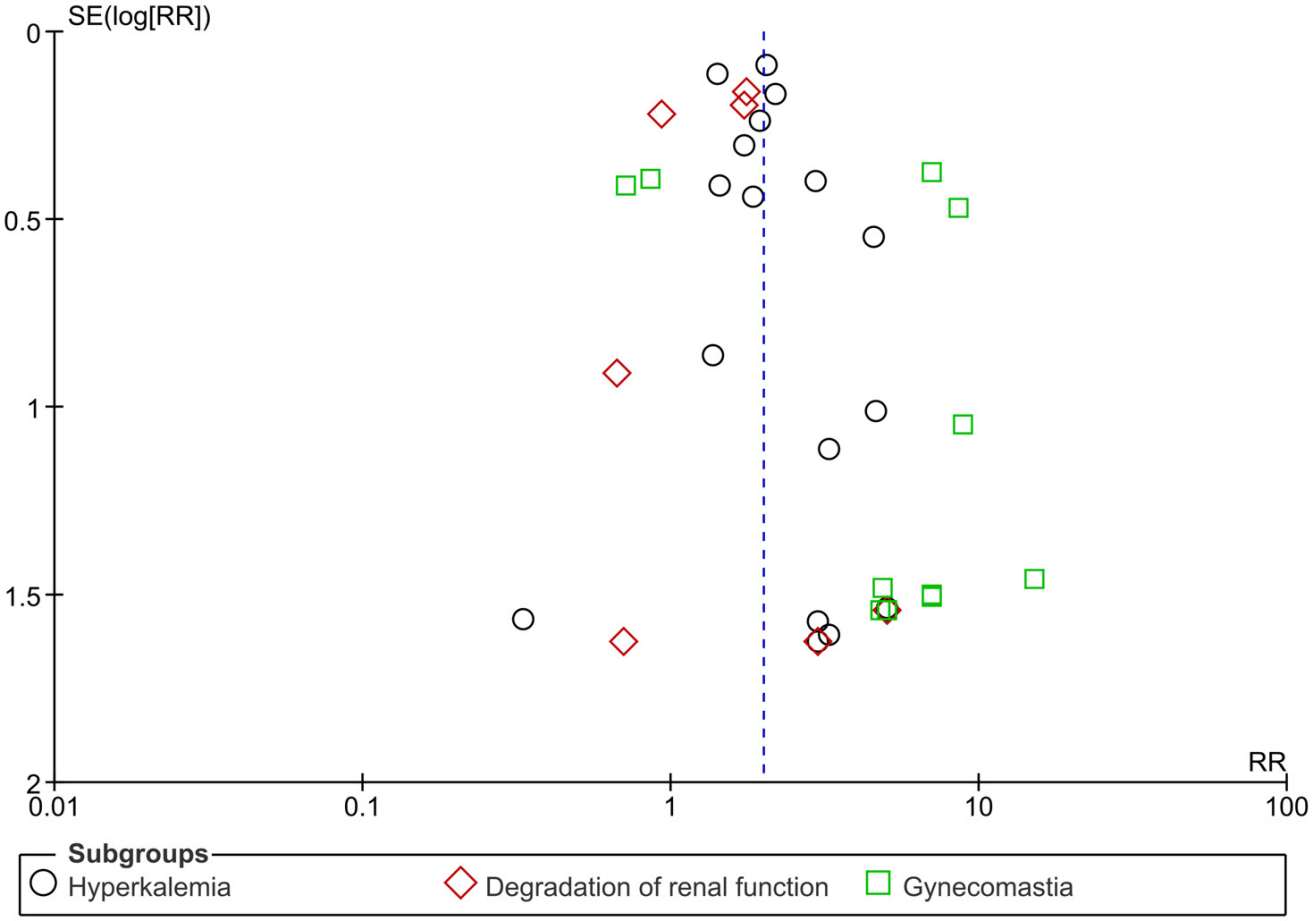
Funnel plot of standard error (log odds ratio) by odds ratio to evaluate publication bias for effect of aldosterone antagonist treatment in inducing common side effects (hyperkalemia, degradation of renal function, gynecomastia) in comparison with placebo/control, in patients with heart failure or myocardial infarction.

Visual analysis of funnel plots suggested the possibility of publication biases in SCD, CV mortality, total/CV hospitalization analyses, with some asymmetries (Figs 5A, 5C and 6A, 6B); this bias was unlikely in two cases: total mortality (Fig 5B) and side effects (Fig 7).

Statistically, potential existence of publication bias was tested by Egger approach, using OR instead of RR for the reason explained in the Method session. For clinical outcome with low incidence (SCD, total/CV mortality, side effects), these two indicators were similar. For example, the SCD prevention effect of AAs estimated by RR was 0.81 (95% CI, 0.71–0.92, p = 0.002) and by OR was 0.80 (95% CI, 0.69–0.92, p = 0.002), both using random effect model. However, the higher the incidence was, the more different these estimators were. For example, for total hospitalization criteria which had the highest incidence (over 40%), intervention effect measured by RR was 0.93 (95% CI, 0.88–0.98, p = 0.008) but by OR was 0.84 (95% CI, 0.72–0.97, p = 0.018), both using random effect model.

Most clinical outcomes in this meta-analysis included at least 10 trials, thus satisfied the recommendations on testing for funnel plot asymmetry, except the primary outcome (SCD). The p-values of Egger tests were 0.21 for SCD, 0.79 for total mortality, 0.17 for CV mortality, 0.13 for total hospitalization, 0.08 for CV hospitalization, 0.23 for hyperkalemia, 0.94 for renal function degradation and 0.29 for gynecomastia, none supporting evidence for publication bias. Of note, regarding both funnel plots & Egger tests, publication biases were not formally assessable for SCD outcome due to the few number of trials included (n = 6).

### Sensitivity analysis

Sensitivity analyses were tested for the biggest trial in each subgroup (among the greatest ones REMINDER [28], TOPCAT [14], EPHESUS [13], RALES [11], EMPHASIS-HF [33]) which had the greatest weight percentages, for eight open label/single blind/not reported design trials if applicable (Cicoira *et al*. [22], Kayrak *et al*. [26], Mak *et al*. [23], Modena *et al*. [27], Vatankulu *et al*. [30], Vizzardi *et al*. 2013 [34], Vizzardi *et al*. 2010 40], Wu *et al*. [24]) and for 11 trials which had no intention to treat analysis (ITTA) if applicable (Boccanelli *et al*. [35], Cicoira *et al*. [22], Deswal *et al*. [36], Di Pasquale *et al*. [25], Kayrak *et al*. [26], Mak *et al*. [23], Taheri *et al*. 2012 [37], Taheri *et al*. 2009 [38], Udelson *et al*. [39], Vizzardi *et al*. 2013 [24], Weir *et al*. [31]) (Table 1). As well, we conducted these analyses only for primary outcome, i.e the preventive effect of AAs on mortality (SCD, total and CV death) in patients with HF or post-MI.

Among all included trials in considering both subgroups, EPHESUS trial [13] contributed the largest weight with relative overall weights of 44.1% for SCD, 34.6% for all-cause mortality and 39.0% for CV mortality analyses. However, when performing a sensitivity test by excluding this trial, no significant differences of RRs were detected for three cases: from (0.81, 95% CI 0.71–0.92, p = 0.002) to (0.81, 95% CI 0.67–0.98, p = 0.03), from (0.82, 95% CI 0.77–0.88, p<0.00001) to (0.80, 95% CI 0.74–0.87, p<0.00001) and from (0.80, 95% CI 0.74–0.87, p<0.00001) to (0.78, 95% CI 0.71–0.86, p<0.00001), respectively.

For patients with HF, the RALES trial [11] had the largest relative weights of 24.6%, 30.8% and 29.4% for these three criteria, respectively. Excluding this trial resulted in no significant difference of estimate effect for SCD analysis: RR (0.81; 95% CI, 0.67–0.98; p = 0.03) changed to (0.82, 95% CI, 0.59–1.14) but the effective estimator turned out non-significant (p = 0.24). The RRs for all-cause and CV mortality changed moderately from (0.81, 95% CI, 0.74–0.88, p <0.00001) to (0.87, 95% CI, 0.77–0.98, p = 0.02) and from (0.79, 95% CI, 0.70–0.89, p = 0.0001) to (0.83, 95% CI, 0.71–0.97, p = 0.02) respectively, with the results remained significant.

In these patients, removing two trials which had no intention-to-treat analysis (ITTA) (Boccanelli *et al*. [35], Taheri *et al*. 2012 [37]) gave no remarkable influence on the AAs’ effect in preventing SCD: RR changed from (0.81; 95% CI, 0.67–0.98; p = 0.03) to (0.83; 95% CI, 0.69–0.99; p = 0.04). The same attempt for three trials (Boccanelli *et al*. [35], Taheri *et al*. 2012 [37], Taheri *et al*. 2009 [38]) resulted in slight changes: RR changed from (0.81, 95% CI, 0.74–0.88, p<0.00001) to (0.81, 95% CI, 0.75–0.88, p <0.00001) and from (0.79, 95% CI, 0.70–0.89, p = 0.0001) to (0.79, 95% CI, 0.70–0.90, p = 0.0004) in case of total/CV mortality, respectively.

Open or single blind trials in HF subgroup were also excluded for sensitivity analyses (applicable for total and CV mortality analyses). Removing the three trials Cicoira *et al*. [22], Mak *et al*. [23], Vizzardi *et al*. 2013 [34] for total mortality and removing the trial of Vizzardi *et al*. 2013 [34] for CV mortality resulted in slight changes: RR changed from (0.81, 95% CI, 0.74–0.88, p<0.00001) to (0.81, 95% CI, 0.73–0.91, p = 0.0004) and from (0.79, 95% CI, 0.70–0.89, p = 0.0001) to (0.83, 95% CI, 0.74–0.94, p = 0.003), respectively.

In those with MI, the EPHESUS trial [13] was the only for SCD prevention analysis. This trial occupied the greatest relative overall weights of 34.6% and 39.0% in case of total and CV mortality, respectively. Removing this trial returned significant changes of RRs from (0.85, 95% CI, 0.76–0.95, p = 0.003) to (0.71, 95% CI 0.48–1.05, p = 0.09) and from (0.83, 95% CI, 0.74–0.94, p = 0.003) to (0.71, 95% CI 0.43–1.18, p = 0.19), respectively.

For total mortality analysis, there was only one trial without ITTA (Weir *et al*. [31]) presented in the MI subgroup and removing this trial had likely no impact on RR: from (0.85, 95% CI, 0.76–0.95, p = 0.003) to (0.85, 95% CI, 0.76–0.94, p = 0.003). Similarly, when three open design trials (Kayrak *et al*. [26], Modena *et al*. [27], Wu *et al*. [24]) were removed, only slight influences on the final effect were observed: RR changed from (0.85, 95% CI, 0.76–0.95, p = 0.003) to (0.83, 95% CI 0.77–0.88, p = 0.006). No trial without ITTA or with single-blind/open design involved MI patients was included for CV mortality analysis.

For SCD, all the included trials concerned HF patients with reduced LVEF, except TOPCAT trial [14] which recruited HF patients with preserved LVEF. Removing this trial resulted in slight change for treatment effect: RR from (0.81, 95% CI, 0.71–0.92, p = 0.002) to (0.78, 95% CI 0.67–0.90, p = 0.0006 and the heterogeneity remained likely absent (both I^2^ = 0%).

## Discussion

In our meta-analysis, we evaluated the efficacy of AAs in reducing mortality (SCD, overall/CV death) and hospitalization rate, as well as their toxicity via the common side effects in 19,333 patients with HF or post-MI from 25 trials. Our findings demonstrated the effectiveness of AAs in preventing SCD, all-cause mortality and CV mortality, yet a double rate of three studied adverse effects in these patients.

The cardio-protective effect of AAs is quite well proven in literature for CV protection [40]. Some of the proposed mechanisms of action in HF of AAs include (i) inhibition of myocardial and vascular remodeling, (ii) blood pressure reduction, (iii) decreased collagen deposition, (iv) decreased myocardial stiffness, (v) prevention of hypokalemia and arrhythmia, (vi) modulation of nitric oxide synthesis, and (vii) immunomodulation [41]. For instance, the meta-analysis of Li *et al*. [42] demonstrated beneficial effects of AAs on the reversal of cardiac remodeling and improvement of left ventricular function. Another quantified AAs’ positive effect on ejection fraction (EF) and functional capacity improvement in different HF functional classes [43].

The RALES trial [11], published in 1999 was the first big study concerning AAs’ effect that recommended this treatment which significantly decreased mortality rate (SCD, all cause and CV death) as well as CV hospitalization rate in patients with severe chronic HF (NYHA III to IV). Next, in 2003, the EPHESUS trial [13] re-confirmed the role of AAs for the same outcomes in patients with AMI complicated by left ventricular dysfunction. This therapy was thus limited to patients with severe HF or those with HF following MI until the publication of EMPHASIS-HF trial [12] in 2011, which reported additional beneficial evidence for AAs use in mild-to-moderate HF (NYHA II), regarding the same clinical criteria. However, the current TOPCAT trial [14] finished in 2014 showed only a significant lower incidence of cardiac hospitalization in those treated by spironolactone vs. placebo, but not for total deaths and all-cause hospitalization, in patients with HF and preserved EF. Sensitivity analysis with this trial suggested that the treatment effect of AAs was likely similar in HF patients with reduced or preserved EF for SCD prevention.

The work of Ezekowitz *et al*. [44] evaluated the effect of aldosterone blockade on left ventricular dysfunction in HF and post-MI participants and reported a significant reduction in overall mortality of 20% (RR 0.80, 95% CI, 0.74–0.87, p<0.00001). That of Hu *et al*. [45], which showed a 21% (RR 0.79, 95% CI 0.66–0.95, p = 0.65) decrease for overall mortality and a 38% (RR 0.62, 95%, CI 0.52–0.74, p = 0.54) decrease for cardiac re-hospitalization by the use of AAs in patients with mild to moderate chronic HF (NYHA I to II). Another current meta-analysis of Bapoje *et al*. [46] that included 8 RCTs, concluded a 23% reduction (OR 0.77; 95% CI, 0.66–0.89; p = 0.001) of SCD in patients with a left ventricular systolic dysfunction of ≤ 45%, treated with AAs. On the contrary, the most recent meta-analysis of Chen *et al*. [47] in 2015 did not observe any all-cause mortality benefit, yet a reduced CV hospitalization rate (RR 0.83; 95% CI; 0.70 to 0.98), in patients with either HF or MI and preserved EF by AA treatment. Our meta-analysis, included MI/ HF patients with both preserved and primarily reduced EF, approved the positive effect of AAs in preventing all considered outcomes: SCD (RR 0.81; 95% CI, 0.71–0.92; p = 0.002), all-cause mortality (RR 0.82; 95% CI, 0.77–0.88, p < 0.0001), CV mortality (RR 0.80; 95% CI, 0.74–0.87, p<0.0001), all–cause hospitalization (RR 0.93; 95% CI, 0.88–0.98; p = 0.008) and CV hospitalization (RR 0.82, 95% CI, 0.72–0.92; p = 0.001) in patients with HF or post MI.

In terms of security, our work demonstrated a doubled rate of common adverse reactions (hyperkalemia, worsening renal function and gynecomastia) in those receiving AAs vs. control or placebo (RR 1.99, 95% CI, 1.64–2.41; p<0.00001). These findings agreed with the results of currently conducted analyses by Clark *et al*. [48] for renal function insufficiency, or by Rossignol *et al*. [49] for hyperkalemia and renal function degradation.

In 2013, a systematic study [50] of conventional HF therapies, including angiotensin-converting enzyme inhibitor (ACEI), angiotensin receptor blocker (ARB), direct renin inhibitor (DRI), and AA compared their effects (on prevention of total death, CV death, non-fatal MI, HF hospitalization and composite of CV death or HF hospitalization) and their safety (on hyperkalemia, hypotension, renal failure). By risk-benefit ratio comparison, this review favored the administration of AA over ARB or DRI, despite its 110% generated increase in hyperkalemia. Likewise, higher proportion of developed hyperkalemia and higher rate of hospitalization for hyperkalemia by AAs in HF patients were recorded in RALES trial, especially in combined use of AAs with either ACEIs or ARBs [51]. Moreover, the benefit of AAs on morbi-mortality prevention seems to overweigh its side-effects, i.e. the reduction in mortality associated with the use of AA was significantly greater than its use complications. Our work estimated numbers of 83, 27 and 18 HF patients need to be treated with AAs to prevent one SCD, one all-cause death and one CV death in one year, respectively. For patients with MI, the corresponding numbers needed to treat (NNT) were 84, 48 and 48, respectively. Considering both patient groups, the estimated NNTs were 83, 34 and 35, respectively. As well, the number needed to harm i.e the number of patients treated on average to have one who suffers at least one of the three common side effects studied, was 77.

Noticeably, focusing on SCD prevention, while AAs help to reduce CV risk factors thus prevent CV accidents including SCD, paradoxically, their side effects of hyperkalemia may induce this accident from cardiac arrhythmia [52]. By this point, a study [53] proved that AAs were independently associated with increased rates of total mortality (hazard ratio HR 1.4; 95% CI 1.1–1.8; P = 0.005), of CV mortality (HR 1.4; 95% CI 1.1–1.9; P = 0.009) and a doubled incidence of SCD (HR 2.0; 95% CI 1.3, 3.0; P = 0.001) in patients with atrial fibrillation and HF. This implied a careful examination of risk/benefit ratio for each individual patient before the prescription of this treatment.

Based on our comprehensive and meticulous search strategy, we believe that we have identified all existing studies that met our inclusion criteria, hence yielding robust results. However, certain limitations should be considered when interpreting these outcomes. For instance, publication bias was not reliably assessed (though seemly negative) for the most important outcome (SCD) when less than 10 studies were included for pooled analyses by funnel plot (Fig 5A) or Egger test.

In summary, to gain the maximum benefit from AAs and reduce possible complications, it is legitimate to individualize and closely monitor their use. For examples, risk-benefit balance should be carefully considered before using AAs in patients with severe renal insufficiency. Also, other factors such as time of treatment initiation [54] and cost difference between AA agents [55] should be taken into account to optimize this therapy.

## Conclusion

Our meta-analysis demonstrates that AA treatment may provide beneficial effects on the prevention of SCD, as well as all-cause and CV mortality, for selected patients with HF with altered left ventricular function or after a MI. Nevertheless, careful consideration before prescribing should be given simultaneously to the therapeutic benefit and the overall safety profile of this medication.

## Supporting Information

S1 App Search strategies in details for the meta-analysis. (DOCX)

S2 App PRISMA checklist for the meta-analysis. (DOC)

S3 App Kappa statistic for data extraction between two independent reviewers of the meta-analysis. (DOCX)

S1 Fig Risk of bias graph: review authors' judgements about each risk of bias item presented as percentages across all included studies. (EPS)

S1 Table Quality assessment of eligible trials comparing aldosterone antagonists with placebo or control. (DOCX)

## Abbreviations

AA: Aldosterone Antagonist
(A)MI: (Acute) Myocardial Infarction
ACEI: Angiotensin-Converting Enzyme Inhibitor
ARB: Angiotensin Receptor Blocker
DRI: Direct Renin Inhibitor
HF: Heart Failure
(LV)EF: (Left Ventricular) Ejection Fraction
LVSD: Left Ventricular Systolic Dysfunction
NYHA: New York Heath Association
RAAS: Renin-Angiotensin Aldosterone hormone System
RCT: Randomized Controlled Trial
SCD: Sudden Cardiac Death
