# Supplementary material for: Impact of Aldosterone Antagonists on Sudden Cardiac Death Prevention in Heart Failure and Post-Myocardial Infarction Patients: A Systematic Review and Meta-Analysis of Randomized Controlled Trials
Source: PLoS One. 2016 Feb 18;11(2):e0145958. doi: 10.1371/journal.pone.0145958 (PMC4758660; doi:10.1371/journal.pone.0145958)
Supplement: S1 App — (DOCX) [file pone.0145958.s001.docx]

S1 Appendix 1_Search strategies in details

# Medline Pubmed

(mineralocorticoid antagonist OR Mineralocorticoid Receptor Antagonists OR antialdosterone OR aldosterone receptor antagonist OR aldosterone receptor blockade OR aldosterone receptor blocker OR aldosterone receptor blocking agents OR canrenoate OR potassium canrenoate OR canrenone OR canrenoic acid OR spironolactone OR eplerenone OR rn 52-01-7 OR rn 107724-20-9 OR aldactone OR ispra) AND (cardiovascular disease OR heart disease OR heart failure OR myocardial infarction OR post acute myocardial infarction)

# Embase

('aldosterone receptor antagonist' OR 'aldosterone receptor blockade' OR 'aldosterone receptor blocker' OR 'aldosterone receptor blocking agents' OR 'canrenoate' OR 'potassium canrenoate' OR 'canrenone' OR 'canrenoic acid' OR 'spironolactone' OR 'eplerenone' OR 'rn 52-01-7' OR 'rn 107724-20-9' OR 'aldactone' OR 'ispra') AND ('cardiovascular disease' OR 'heart disease' OR 'heart failure' OR 'myocardial infarction' OR 'post acute myocardial infarction') AND ([controlled clinical trial]/lim OR [randomized controlled trial]/lim OR [meta analysis]/lim) AND [humans]/lim)

# Cochrane library

(mineralocorticoid antagonist OR Mineralocorticoid Receptor Antagonists OR antialdosterone OR aldosterone receptor antagonist OR aldosterone receptor blockade OR aldosterone receptor blocker OR aldosterone receptor blocking agents OR canrenoate OR potassium canrenoate OR canrenone OR canrenoic acid OR spironolactone OR eplerenone OR rn 52-01-7 OR rn 107724-20-9 OR aldactone OR ispra) AND (cardiovascular disease OR heart disease OR heart failure OR myocardial infarction OR post acute myocardial infarction) AND (RCT OR randomi$ez controlled trial* OR clinical trial* OR meta analys$s)

# Web of science

You searched for: **TOPIC:** ((mineralocorticoid antagonist OR Mineralocorticoid Receptor Antagonists OR antialdosterone OR aldosterone receptor antagonist OR aldosterone receptor blockade OR aldosterone receptor blocker OR aldosterone receptor blocking agents OR canrenoate OR potassium canrenoate OR canrenone OR canrenoic acid OR spironolactone OR eplerenone OR rn 52-01-7 OR rn 107724-20-9 OR aldactone OR ispra) AND (cardiovascular disease OR heart disease OR heart failure OR myocardial infarction OR post acute myocardial infarction) AND (RCT OR randomi$ez controlled trial* OR clinical trial* OR meta analys$s))

**Refined by:** **RESEARCH DOMAINS:** (SCIENCE TECHNOLOGY ) AND **RESEARCH AREAS:** (CARDIOVASCULAR SYSTEM CARDIOLOGY OR PHARMACOLOGY PHARMACY OR ENDOCRINOLOGY METABOLISM OR GENERAL INTERNAL MEDICINE OR TOXICOLOGY )

**Timespan:** All years.

Search language=Auto

# clinicaltrials.gov

(«aldosterone receptor antagonist» OR «aldosterone receptor blockade» OR «aldosterone receptor blocker» OR «aldosterone receptor blocking agents» OR «canrenoate» OR «potassium canrenoate» OR «canrenone» OR «canrenoic acid» OR «spironolactone» OR «eplerenone» OR «rn 52-01-7» OR «rn 107724-20-9» OR «aldactone» OR «ispra») AND («cardiovascular disease» OR «heart disease» OR «heart failure» OR «myocardial infarction» OR «post acute myocardial infarction»)

# Others

<https://www.clinicaltrialsregister.eu>

<http://www.trialdetails.com/search>

aldosterone AND (heart failure OR myocardial infarction)
