# Supplementary material for: Impact of Aldosterone Antagonists on Sudden Cardiac Death Prevention in Heart Failure and Post-Myocardial Infarction Patients: A Systematic Review and Meta-Analysis of Randomized Controlled Trials
Source: PLoS One. 2016 Feb 18;11(2):e0145958. doi: 10.1371/journal.pone.0145958 (PMC4758660; doi:10.1371/journal.pone.0145958)
Supplement: S3 App — (DOCX) [file pone.0145958.s003.docx]

S3 Appendix_Kappa statistic

***Table A2. Kappa statistic for data extraction between two independent reviewers of this meta-analysis***

|  |  | Review author 1 (HHL) | | |  |
| --- | --- | --- | --- | --- | --- |
|  |  | Include | Exclude | Unsure | Total |
| Review author 2 (MM) | Include | *a = 25* | *b = 1* | *c = 0* | I_1_ = 26 |
|  | Exclude | *d = 2* | *e = 0* | *f = 0* | E_1_ = 2 |
|  | Unsure | *g = 0* | *h = 0* | *i = 6* | U_1_ = 6 |
|  | Total | I_2_ = 27 | E_2_ = 1 | U_2_ = 6 | K = 34 |

P_0_ = 31/34 = 0.912

P_E_ = 0.64

**Kappa = 0.75 (IC 95% CI, 0.49-1.02; p=0.0005)**

(Good as suggested by (Altman, 1991)*) with


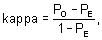


where


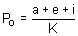


is the proportion of studies for which there was agreement, and


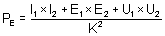


is the proportion of studies in which one would expect there to be agreement by chance alone.

(*)Altman, D.G.: Practical Statistics for Medical Students. Chapman and Hall, London (1991)
