# Supplementary material for: Impact of Aldosterone Antagonists on Sudden Cardiac Death Prevention in Heart Failure and Post-Myocardial Infarction Patients: A Systematic Review and Meta-Analysis of Randomized Controlled Trials
Source: PLoS One. 2016 Feb 18;11(2):e0145958. doi: 10.1371/journal.pone.0145958 (PMC4758660; doi:10.1371/journal.pone.0145958)
Supplement: S1 Table — (DOCX) [file pone.0145958.s005.docx]

# S1 Table. Quality assessment of eligible trials comparing aldosterone antagonists with placebo or control

| **Studies** | **Random sequence generation** | **Allocation concealment** | **Blinding of participants, investigators** | **Blinding of outcome assessment** | **Incomplete outcome data** | **Selective outcome reporting** | **Other threats to validity** |
| --- | --- | --- | --- | --- | --- | --- | --- |
| *Boccanelli et al. 2009 (AREA-in-HF)* [22] | LR | LR | LR | LR | LR | LR | LR |
| *Chan et al. 2007* [23] | LR | LR | LR | LR | LR | LR | UR† |
| *Cicoira et al. 2002* [24] | LR | LR | HR* | LR | LR | LR | LR |
| *Deswal et al. 2011*  *(RAAM-PEF)* [25] | LR | LR | LR | LR | LR | LR | UR† |
| *Di Pasquale et al. 2005* [26] | LR | LR | LR | LR | LR | LR | LR |
| *Edelmann et al. 2013* [27] | LR | LR | LR | LR | LR | LR | LR |
| *Gao et al. 2007* [28] | LR | LR | LR | LR | LR | LR | LR |
| *Kayrak et al. 2010* [29] | LR | LR | HR* | LR | LR | LR | LR |
| *Mak et al. 2009* [30] | LR | LR | HR* | LR | LR | LR | UR† |
| *Modena et al. 2001* [31] | LR | LR | UR†† | LR | LR | LR | UR† |
| *Montalescot et al. 2014 (REMINDER)* [32] | LR | LR | LR | LR | LR | LR | LR |
| *Pitt et al. 2014 (TOPCAT)* [14] | LR | LR | LR | LR | LR | LR | LR |
| *Pitt et al. 2003 (EPHESUS)* [13] | LR | LR | LR | LR | LR | LR | LR |
| *Pitt et al. 1999 (RALES)* [11] | LR | LR | LR | LR | LR | LR | LR |
| *Taheri et al. 2012* [33] | LR | LR | LR | UR†† | LR | LR | UR† |
| *Taheri et al. 2009* [34] | LR | LR | LR | LR | LR | LR | UR† |
| *The RALES Investigators* [35] | LR | LR | LR | LR | LR | LR | LR |
| *Udelson et al. 2010* [36] | LR | LR | LR | LR | LR | LR | LR |
| *Uzunhasan 2009* [37] | LR | LR | LR | UR†† | LR | LR | HR‡ |
| *Vatankulu et al. 2013* [38] | LR | LR | HR* | LR | LR | LR | LR |
| *Vizzardi et al. 2013* [39] | LR | LR | UR** | LR | LR | LR | LR |
| *Vizzardi et al. 2010* [40] | LR | LR | UR** | LR | LR | LR | LR |
| *Weir et al. 2009* [41] | LR | LR | LR | LR | LR | LR | LR |
| *Wu et al. 2013* [42] | LR | LR | HR* | UR†† | LR | LR | LR |
| *Zannad et al. 2011 (EMPHASIS-HF)* [43] | LR | LR | LR | LR | LR | LR | LR |

LR: low risk; HR: high risk; UR: Unclear risk

* absence of placebo;

** single-blind fashion;

† small sample size(< 30 patients/arm)

†† clear description of criteria not available
